# Spatial clustering of zero dose children aged 12 to 59 months across 33 countries in sub-Saharan Africa: A multiscale geographically weighted regression analysis

**DOI:** 10.1371/journal.pone.0338568

**Published:** 2025-12-10

**Authors:** Chamberline E. Ozigbu, Zhenlong Li, Bankole Olatosi, James W. Hardin, Nicole L. Hair

**Affiliations:** 1 Department of Health Services Policy and Management, Arnold School of Public Health, University of South Carolina, Columbia, South Carolina, United States of America; 2 Geoinformation and Big Data Research Lab, Pennsylvania State University, University Park, Pennsylvania, United States of America; 3 South Carolina SmartState Center for Healthcare Quality, Arnold School of Public Health, University of South Carolina, Columbia, South Carolina, United States of America; 4 Big Data Health Science Center (BDHSC), University of South Carolina, Columbia, South Carolina, United States of America; 5 Department of Epidemiology and Biostatistics, Arnold School of Public Health, University of South Carolina, Columbia, South Carolina, United States of America; University of Wyoming, UNITED STATES OF AMERICA

## Abstract

While prior studies have identified sociodemographic correlates of zero-dose status within populations in sub-Saharan Africa (SSA), few have applied spatial regression techniques to explore geographic variability in these relationships. We aimed to address this gap using data from Demographic and Health Surveys conducted in SSA between 2010 and 2020. Our sample comprised children aged 12–59 months in 33 countries and 329 survey regions. Data were aggregated to the first-level administrative unit prior to analysis. First, using ordinary least squares regression, we documented global relationships between theoretically important sociodemographic characteristics and zero-dose prevalence. Next, we identified patterns, i.e., geographic clustering, of zero-dose prevalence. Finally, using multiscale geographically weighted regression, we described spatial variability in relationships between sociodemographic characteristics and zero-dose prevalence. We detected 27 regions with higher than expected concentrations of zero-dose children. All but one of these hot spots were observed in 7 Western and Central African countries; only 1 was located in an Eastern African country. Regions with higher proportions of mothers with no antenatal care visits were consistently found to have higher rates of zero-dose children. In contrast, relationships between zero-dose prevalence and indicators of religious affiliation, delivery site, maternal age, maternal education, and maternal employment were found to vary locally in terms of their strength and/or direction. Study findings underscore spatial disparities in zero-dose prevalence within SSA and, further, highlight the importance of geographically informed strategies to effectively address immunization gaps. Implementing targeted interventions based on regional sociodemographic dynamics is crucial for achieving comprehensive vaccination coverage in SSA.

## Introduction

According to World Health Organization (WHO) and United Nations Children’s Fund (UNICEF) estimates, in 2022 there were approximately 14 million zero-dose children, i.e., children who had not received a single vaccine dose, residing mainly in 10 low- and middle-income countries (LMIC) [1]. COVID-19 pandemic-related disruptions led to more children missing essential vaccines [2,3]. In response, WHO and UNICEF have set a target of reducing the number of zero-dose children by 50% globally [4]. In alignment with Immunization Agenda 2030 (IA2030) goals, the Gavi 2021−25 (Gavi 5.0) strategy prioritizes equity in immunization service delivery, with a focus on extending services to under-vaccinated and, in particular, zero-dose children [4,5]. Achieving these goals necessitates a comprehensive understanding of the geographical distribution and sociodemographic factors influencing zero-dose populations.

Previous studies of populations in sub-Saharan Africa (SSA) indicate that zero-dose children tend to be less socioeconomically privileged, more likely to reside in rural households, and often raised by mothers with limited to no education [6–9]. Spatial analyses of childhood immunization in SSA have typically been constrained to mapping prevalence in a particular country or locality, or have focused on coverage for a specific vaccine dose or series (with the exception of Arambepola et al.) [10–14]. Few analyses specifically address spatial variability in zero-dose populations.

This study seeks to address this current gap in the existing literature by employing multiscale geographically weighted regression (MGWR) on Demographic and Health Survey (DHS) data from 33 SSA countries. While MGWR has been used to uncover spatial variability in other important public health outcomes [15,16], this approach is yet to be applied in the context of zero-dose populations. The overarching research objectives are twofold: (1) To examine the geographic distribution and spatial clusters of zero-dose children aged 12–59 months across 329 regions in SSA and (2) To examine spatial variability in associations between sociodemographic characteristics and the prevalence of zero-dose children in SSA.

## Methods

### Data

Data were obtained with permission from the DHS program [17]. The DHS has been in existence for over three decades, with more than 400 surveys conducted across 90 countries during this period [18,19]. The DHS are nationally representative, cross-sectional, household surveys conducted every 5 years using a standardized approach that is designed to allow for comparison across countries [19]. Among other topics, the DHS collect information on childhood immunization, reproductive health, maternal and child health, malaria, infant and child mortality, and HIV/AIDS [19]. DHS data also include sociodemographic characteristics of the child, mother, and household. The DHS program uses a stratified two-stage cluster probabilistic sampling design, with response rates typically exceeding 90% [19,20]. In the first stage, the enumeration areas (EA) are selected from the primary sampling units (PSU) drawn from census files, with the probability of choosing a unit comparative to its size within each stratum. The second stage, involves selecting a sample of households by equal probability systematic sampling in each EA [19,20]. Details about the DHS program and its sampling design has been published elsewhere [19,20]. Countries with a standard DHS conducted between 2010–2020 were selected for the study. This yielded a total of 33 eligible countries with 329 DHS survey regions (**Table 1**). In total, the analytic sample included 133,739 children aged 12–59 months at the time of the survey.

**Table 1:** Country, survey year, weighted sample size and number of DHS survey regions in this analysis.

Country	Survey Year	Sample Size (Weighted)	Number of Survey Regions
Angola	2015–2016	5,333	18
Burkina Faso	2010	5,274	13
Benin	2017–2018	4,747	12
Burundi	2016–2017	4,954	18
Congo DRC	2013–2014	6,502	11
Congo	2011–2012	3,364	11
Cote d’Ivoire	2011–2012	2,554	10
Cameroun	2018	3,376	12
Ethiopia	2016	3,770	11
Gabon	2012	2,558	10
Ghana	2014	2,103	10
Gambia	2019–2020	2,981	8
Guinea	2018	2,622	8
Kenya	2014	7,262	8
Comoros	2012	2,093	3
Liberia	2019–2020	1,971	5
Lesotho	2014	984	10
Mali	2018	3,631	9
Malawi	2015–2016	6,422	3
Mozambique	2011	7,130	11
Nigeria	2018	11,732	6
Niger	2012	4,263	8
Namibia	2013	1,369	13
Rwanda	2014–2015	2,807	5
Sierra Leone	2019	3,534	5
Senegal	2010–2011	3,234	14
Chad	2014–2015	8,141	21
Togo	2013–2014	2,536	5
Tanzania	2015–2016	3,847	29
Uganda	2016	5,653	4
South Africa	2016	1,260	9
Zambia	2018	3,723	9
Zimbabwe	2015	2,009	10
**Total**		**133,739**	**329**

### Data preparation

DHS data were aggregated to first-level administrative units prior to analysis. The first-level administrative units used in our analysis refer to the primary subnational divisions within a country, such as states, provinces, or regions, depending on the country-specific administrative structure. These units are the highest subnational level at which DHS data are typically disaggregated for analysis and reporting. We obtained the first-level subdivision boundary of sub-Saharan Africa in shapefile format from the Database of Global Administrative Areas [21]. Geographically referenced data for zero-dose children was extracted for each first-level administrative unit. The attributes table representing relevant variables was added and joined to the shapefile showing countries in SSA. The proportion of zero-dose children for each administrative unit were used to generate choropleth maps.

### Study design

We conducted a secondary analysis of existing data on childhood immunization using a pooled cross-sectional study design. The study protocol was reviewed by the institutional review board at the University of South Carolina. The study was determined to meet the Not Human Subject criteria set forth by the Code of Federal Regulations (45 CFR 46). Since this study involved secondary analysis of existing data sources and no more than minimal risk, consent was waived for the study.

### Outcome variable

The outcome variable is the proportion of children aged 12–59 months with zero-dose vaccination status (i.e., children who have not received a single vaccine dose).

### Explanatory variables

The selection of explanatory variables was guided by the literature and availability in the DHS dataset [22–24]. We considered child’s sex (proportion of children who are female), child’s birth weight (proportion of children with low birth weight, i.e., birth weight <2500 grams), mother’s age (proportion of mothers aged 15–19 years), mother’s education (proportion of mothers with no education), mother’s employment status (proportion of mothers not currently working), media access (proportion of children in households with no media access, i.e., no regular exposure to television, radio or newspapers), mother’s marital status (proportion of mothers who are unmarried), antenatal care utilization (proportion of mothers who reported no antenatal care [ANC] visits during their pregnancy), place of delivery (proportion of mothers who delivered at home), place of residence (proportion of children living in rural areas), wealth index (proportion of children living in the poorest households, i.e., lowest quintile of the DHS wealth index) and religion (proportion of children with Muslim mothers). To ensure representativeness of the sample at the first-level administrative unit we utilized weighted proportions for which the sampling weights are normalized values from the DHS datafile [25].

### Statistical analysis

#### Global and local spatial autocorrelation of zero-dose.

Spatial autocorrelation measures the degree of similarity between neighboring regions for a specific measure. This can be assessed either globally (all measured points in the data) or locally (only those measures within a certain distance). We utilized Moran’s *I* to summarize spatial autocorrelation of location-specific rates of zero-dose children and then used regression analysis to examine the factors that are associated with the spatial clustering of zero-dose children.

The global Moran’s *I* statistic was used to measure global spatial autocorrelation. While the global Moran’s *I* measures the degree of spatial autocorrelation across study areas, it lacks precision to show where the values of the variables are spatially correlated. The local indicators of spatial association (LISA) method addresses this limitation by measuring the localized spatial autocorrelation within a study area [26]. Thus, this study utilized LISA for Moran’s *I* to measure local spatial autocorrelation [26,27]. The global Moran’s *I* index ranges from −1–1 and is a single estimate of spatial autocorrelation. Values close to zero indicate the absence of a spatial association or dependence (i.e., a random distribution) [28]. However, values close to +1 indicates strong clustering and values close to −1 indicates strong spatial dispersion [28].

The LISA analysis was used to produce a spatial layer made up of 4 types of spatial clusters that are statistically significant across neighboring regions described as high-high (hot spots), low-low (cold spots), low-high (spatial outliers), and high-low (spatial outliers) [26]. “High-high” and “low-low” clusters show positive spatial autocorrelations (i.e., regions with high rates of zero-dose children that share borders with neighbors that also have high rates of zero-dose children or regions with low rates of zero-dose children that share borders with neighbors that also have low rates of zero-dose children, respectively). “High-low” and “Low-high” clusters indicate negative autocorrelations (i.e., regions with high rates of zero-dose children that share borders with neighbors that have low rates of zero-dose children or regions with low rates of zero-dose children that share borders with neighbors that have high rates of zero-dose children, respectively). We establish spatial contiguity using Rook second order for spatial autocorrelation [29].

#### Regression analysis.

We utilized OLS regression to evaluate global relationships between the prevalence of zero-dose children and sociodemographic predictors and a multiscale geographically weighted regression (MGWR) analysis to allow those relationships to vary locally.

#### Ordinary least squares model.

First, a traditional OLS regression model was used to define a baseline model. This global model is defined as


$$y_{i}=\;\beta_{\text{0}}+\beta_{X_{i}}+\;\varepsilon_{i}$$ (1)

where $y_{i}$ is the proportion of children who are zero-dose in region i; $\beta_{\text{0}}$ is the intercept; β is the vector of the estimated coefficients of covariates; $X_{i}$ is the vector of selected covariates, and $\varepsilon_{i}$ is the error term in the model estimates.

We calculated the spatial autocorrelation and inferred that the Moran’s *I* for our sample indicated positive spatial autocorrelation. We further assessed the spatial autocorrelation of the residuals from the OLS model which show strong spatial autocorrelation (regions close to each other tend to have similar residuals) [30]. Thus, the OLS regression assumption of independence was violated. The OLS model could only account for a global effect and does not capture the spatial non-stationarity.

#### Multiscale geographically weighted regression model.

To address these violations of the linear regression assumptions multiscale geographically weighted regression (MGWR), a variant of geographically weighted regression (GWR), was utilized to capture spatial heterogeneity and allow relationships between the proportion of zero-dose children and covariates to vary locally using separate bandwidths for each covariate [31]. Although GWR can capture spatial heterogeneity, it applies a uniform spatial scale for all covariates which may potentially bias results by either underestimating or amplifying the influence of each covariate [31]. However, MGWR uses an iterative back-fitting algorithm to assess the goodness of fit for each covariate [31]. The model is defined as


$$y_{i}=\sum_{j=\text{1}}^{M}\beta_{bwj}X_{ij}+\varepsilon_{i}$$ (2)

where $y_{i}$ is the proportion of children who are zero-dose in region *i*; $\beta_{bwj}$ is the estimation of coefficient for region *i* in which *bwj* is the jth optimal bandwidth; $X_{ij}$ is the jth covariate at region *i*; M is the number of covariates, and $\varepsilon_{i}$ is the error term in the model estimates.

Models were calibrated using adaptive bi-square spatial kernel for weighting the data in the golden section bandwidth search [32]. Independent variables were standardized to ensure coefficients were comparable in terms of relative influence.

#### Model evaluation.

All regression models were specified using a consistent set of predictor variables, as defined in the “Explanatory Variables” section. Model performance was compared using Akaike information criterion (AIC), corrected AIC (AICc), adjusted R-squared, and log-likelihood values. A model with lower AIC and AICc values and higher adjusted R-squared and log-likelihood values was preferred, as it explained more of the variation in zero-dose prevalence across regions. We assessed multicollinearity in our final regression model using condition numbers (<30) and variance composition proportions (<0.5) and confirmed that there was no evidence of substantial multicollinearity. We utilized MGWR software, version 2.2.1 to fit all the models, and QGIS software version 3.24.3 to generate the maps. A p-value <0.05 was considered statistically significant.

## Results

### Descriptive results

**Fig 1:**
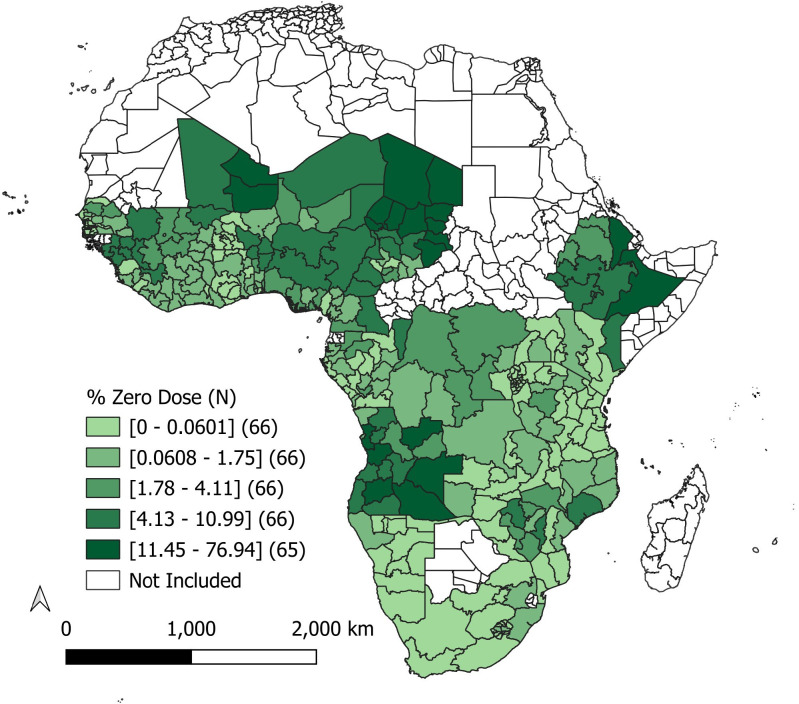
Prevalence of zero-dose children across SSA. Five categories defined by quintiles. First-level subdivision boundary shapefiles were downloaded from the GADM website (https://gadm.org/).

**Table 2** shows descriptive statistics for the outcome of interest and explanatory variables. The prevalence of zero-dose children varied considerably across the 33 SSA countries included in our sample, from 0% in Namibia (Karas region) to 76.9% in Chad (Borkou/Tibesti region) (**Fig 1** and S1 Fig). The mean prevalence of zero-dose status among children aged 12–59 months was 6.8% (SD: 10.4%). Choropleth maps showing the spatial distribution of explanatory variables are available in S2 Fig.

**Table 2:** Descriptive analysis of outcome and explanatory variables.

Variables	Mean (SD)
**Outcome Variable**	
Proportion of children who are zero-dose	6.8 (10.4)
**Explanatory Variables**	
Proportion of children who are female	5.0 (3.7)
Proportion of children with low birth weight^a^	6.4 (4.0)
Proportion of mothers aged 15–19 years	5.7 (3.4)
Proportion of mothers with no education	3.8 (3.1)
Proportion of mothers not currently working	4.0 (2.4)
Proportion of children in households with no media access^b^	3.9 (1.9)
Proportion of mothers who are unmarried	1.6 (1.4)
Proportion of mothers who reported no ANC visits during their pregnancy	8.8 (10.2)
Proportion of mothers who delivered at home	3.2 (2.5)
Proportion of children living in rural areas	6.7 (2.8)
Proportion of children living in the poorest households^c^	2.4 (1.8)
Proportion of children with Muslim mothers	2.8 (3.8)

^a^ Low birth weight defined as birth weight < 2,500 grams.

^b^ No media access defined as no regular exposure to television, radio or newspapers.

^c^ Poorest households defined to include households in the lowest quintile of the DHS wealth index.

### Cluster analyses

**Fig 2:**
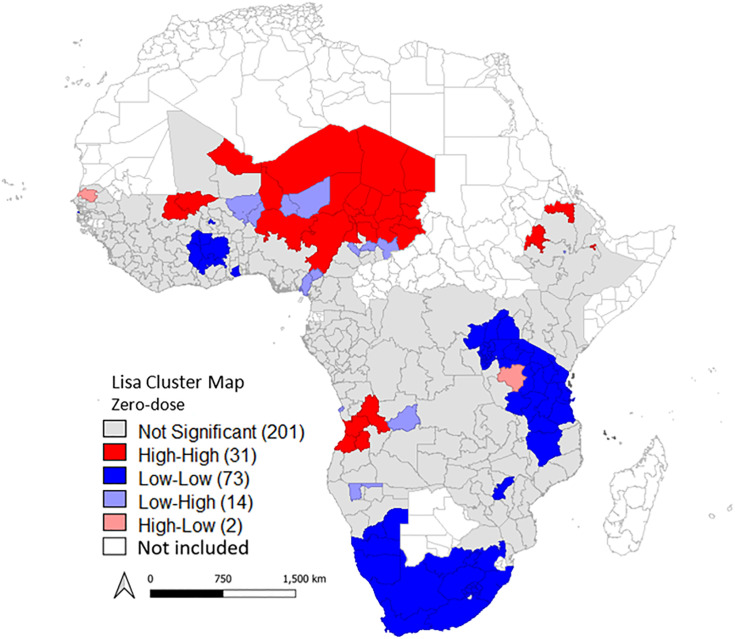
Univariate local indicators of spatial association (LISA) cluster map of zero-dose rates. First-level subdivision boundary shapefiles were downloaded from the GADM website (https://gadm.org/).

We observed significant positive spatial autocorrelation in the prevalence of zero-dose children across SSA countries with a global Moran’s *I* index of 0.33 (p-value = 0.001). The LISA cluster map (**Fig 2**) shows high-high clusters (neighboring regions with high rates of zero-dose children clustered to form hot-spots) involving 31 regions in 7 SSA countries (21.2%). Most of the high-high clusters were located in six Western and Central African countries (Angola, Cameroon, Chad, Mali, Niger, and Nigeria), with only one country in Eastern Africa (Ethiopia) observed to have high-high clusters. The cold-cold clusters (cold-spots) were formed by the clustering of 73 neighboring regions from 16 SSA countries (48.4%) with low rates of zero-dose children. The cold-cold clusters shared borders with Burundi, Congo DRC, Gambia, Ghana, Lesotho, Namibia, Rwanda, Tanzania, Uganda, South Africa, Zimbabwe, Mozambique, Burkina-Faso, Togo, Côte d’Ivoire and Kenya. Adjacent to these hot- and cold-spots were outliers: low-high and high-low clusters, respectively. The low-high clusters highlighted 11 SSA countries (Angola, Cameroon, Chad, Guinea, Mozambique, Namibia, Niger, Sierra Leone, South Africa, Zambia, and Zimbabwe; 33.3%) with significantly low zero-dose rates that were neighbors to 17 regions with significantly high zero-dose rates. High-low clusters were observed in 6 regions from 3 SSA countries: Burundi, South Africa, and Tanzania.

### Global model with OLS

We utilized OLS regression to evaluate the relationship between the prevalence of zero-dose children and sociodemographic predictors. **Table 3** presents the coefficient, standard error, t-statistic, and p-value for each explanatory variable included in the OLS regression. As shown, the proportions of adolescent mothers, mothers with no media access, women with no antenatal care visit, children whose mother had a home delivery and children living in rural areas were statistically significantly associated with the prevalence of zero-dose children.

**Table 3:** Ordinary least square regression results.

Variable	coefficient	Std. Error	t-statistic	p-value
Intercept	−0.000	0.031	−0.000	1.000
Proportion of children who are female	−0.006	0.031	−0.191	0.849
Proportion of children with low birth weight^a^	−0.041	0.044	0.947	0.343
**Proportion of mothers aged 15–19 years**	**0.104**	**0.035**	**3.019**	**0.003**
Proportion of mothers with no education	0.037	0.058	0.631	0.528
Proportion of mothers not currently working	−0.024	0.036	−0.668	0.504
**Proportion of children in households with no media access** ^b^	**0.176**	**0.047**	**3.717**	**0.000**
Proportion of mothers who are unmarried	−0.083	0.046	−1.813	0.070
**Proportion of mothers who reported no ANC visit during their pregnancy**	**0.589**	**0.045**	**13.073**	**0.000**
**Proportion of mothers who delivered at home**	**0.214**	**0.058**	**3.690**	**0.000**
**Proportion of children living in rural areas**	**−0.162**	**0.046**	**−3.548**	**0.000**
Proportion of children living in the poorest households^c^	−0.008	0.036	−0.232	0.817
Proportion of children with Muslim mothers	0.061	0.053	1.160	0.246

^a^ Low birth weight defined as birth weight < 2,500 grams.

^b^ No media access defined as no regular exposure to television, radio or newspapers.

^c^ Poorest households defined to include households in the lowest quintile of the DHS wealth index.

**Fig 3:**
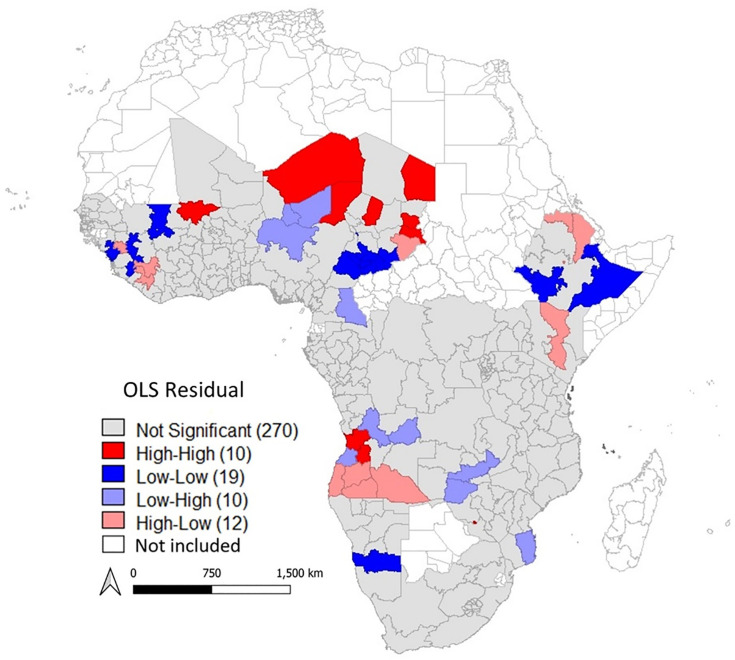
Map of OLS regression residuals. First-level subdivision boundary shapefiles were downloaded from the GADM website (https://gadm.org/).

**Fig 3** shows residuals of the OLS regression model. The global Moran *I* index is 0.060 with a z-score of 7.4421, indicating that the residuals are significantly clustered (spatially autocorrelated) with a p-value of 0.001. The OLS model overestimates the prevalence of zero-dose children in regions of Angola (Cuanza Sul and Huambo), Zimbabwe (Bulawayo), Mali (Mopti), Niger (Agadez and Diffa), Chad (Bahr el Gazel, Ennedi, Ouaddai, and Sila). Also, it underestimates prevalence of zero-dose children in Chad (Longoe-Occidental, Logone-Oriental, Mandoul, Mayo-Kebbi-Est, Mayo-Kebbi-Ouest, Moyen-Chari, Tandjile and Ville de Ndjamena), Namibia (Hardap), Cameroon (Cameroon North), Guinea (Kindia), Mali (Koulikoro) and Ethiopia (Dire Dawa, Somali, Harari, SNPP and Gambela).

### Evaluation metrics of OLS and MGWR models

**Table 4** summarizes evaluation metrics for the OLS and MGWR models. The MGWR model performed better than the OLS model, given that MGWR had smaller AICc and AIC than the OLS, indicating an improved model. Also, the adjusted R-squared in the MGWR was higher than in the OLS, suggesting that 88% of the variability observed in the prevalence of children with zero-dose status is explained by the MGWR compared to OLS model which explains 69% variability. Additionally, the value of log-likelihood in the MGWR model was higher than the OLS model, signifying a better model fit.

**Table 4:** Comparison of MGWR and OLS models of zero-dose rates in SSA.

	OLS Model	MGWR Model
**Model Comparison**		
AICc	562.536	337.163
AIC	559.198	293.920
Adjusted R^2^	0.693	0.883
Log-Likelihood	−266.599	−73.301

AICc = corrected Akaike information criterion; AIC= Akaike information criterion

### Local models with MGWR

**Fig 4:**
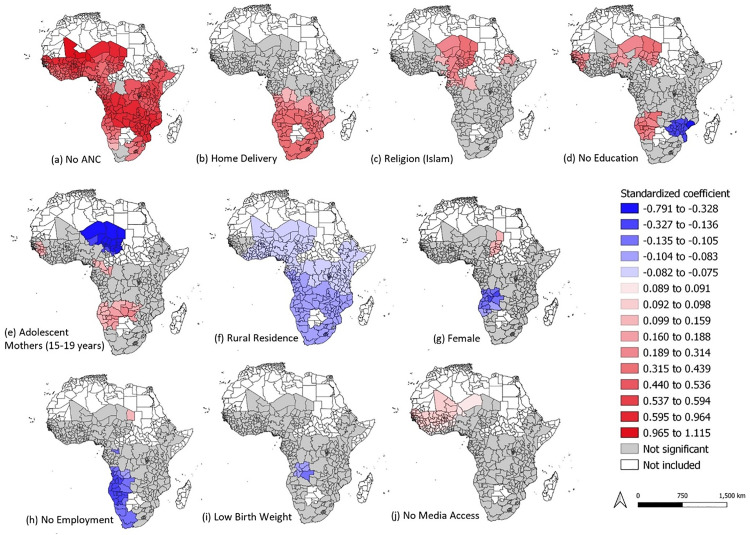
Spatial distribution of the coefficients of variables (standardized). The color scheme is uniform in all the maps, allowing for variables to be comparable in direction and magnitude. Blue corresponds to negative associations, whereas red corresponds to positive associations. First-level subdivision boundary shapefiles were downloaded from the GADM website (https://gadm.org/).

MGWR generates a local model for each first-level administrative unit to capture spatial heterogeneity and allow relationships between the proportion of children with zero-dose status and covariates to vary locally using separate bandwidths for each explanatory variable. To examine and compare the variable coefficients, we utilized map matrix to map all variables using the same color scheme in one map and then classified all the standardized coefficients into seventeen classes. Interesting spatial patterns can be observed across countries and regions of SSA (**Fig 4**).

#### No ANC visit.

There is a positive association between the proportion of children with zero-dose status and the proportion of women with no ANC visits across most countries and regions in SSA (**Fig 4a**). The observed relationship was strongest in regions of Niger (Agadez, Maradi, Tahoua and Tillaberi), Mali (Gao, Mopti, Kayes, Koulikoro, Segou, and Tombouctou), Chad (Sila, Bahr el, Batha, Ennedi, Ouaddai, and Borkou/Tibesti), Congo DRC (Bandundu, Katanga, Kasai-Oriental and Kasai-Occidental), Zambia (Central, Eastern, Southern, Luapula, Lusaka, North-Western, and Western), Mozambique (Cabo Degado, Niassa, Nampula, Sofala, Zambezia, Manica and Tete), and Zimbabwe (Harare, Mashonaland West, Mashonaland East and Mashonaland Central). No significant relationship was detected in just three regions: the DRC equateur region in Congo DRC and the Western Cape and Northern Cape regions in South Africa.

#### Home delivery.

**Fig 4b** shows a significant association between the proportion of children with zero-dose status and the proportion of women with home delivery, particularly in Southern Africa and some regions in East African countries. Weaker but positive associations were observed in Congo DRC (Bandundu, Kasai-Occidental and Katanga), Mozambique (Niassa) and Zambia (Northern). No significant findings were observed for other regions studied.

#### Religion (Islam).

**Fig 4c** shows a positive spatial association between the proportion of children with zero-dose status and the proportion of Muslim mothers. This relationship was observed in some regions within West, Central and East African countries. The relationship was particularly strong in regions of Ethiopia (Tigrai) and Chad (Batha, Ennedi, Sila, Wadi Fira and Ouaddai). Weaker but statistically significant associations were observed in regions of Nigeria (North-West), Niger (Maradi), Gabon (Ogooue-Ivindo, Woleu-Ntem) and Ethiopia (Amhara and Afar). Other regions in SSA did not show any significant findings.

#### No education.

The relationship between the proportion of children with zero-dose status and the proportion of mothers with no education varied across regions in SSA (**Fig 4d**). For instance, some parts of West, Central, South and East African countries show both significant and no significant association. Regions in a few countries, including Chad (Wadi Fira, Borkou/Tibesti, and Ennedi), Angola (Moxico, Cunene), and Namibia (Oshikoto, Oshana, Omusati and Kunene) show a strong positive association. In contrast, in East Africa, there was a strong inverse relationship (blue area), mostly observed in Zimbabwe (all regions), Mozambique (Tete, Sofala, Manica, and Inhambane), Zambia (Zambia Southern, and Lusaka).

#### Adolescent mothers (15–19 years).

The association between the proportion of adolescent mothers and the proportion of children with zero-dose status varies across regions showing both significant (both positive and inverse) and non-significant results (**Fig 4e**). A strong inverse relationship was observed in Chad (across all regions) and in a few regions in Niger (Agadez, Diffa, and Zinder). Conversely, weaker positive associations were found in select regions of Senegal (Kaffrine, Kedougou, Kolda, and Tambacounda), Gambia (Upper River and MacCarthy Island), Guinea (Boke, Mamou, Labe, and Kindia), Congo (Sangha and Likouala), Zambia (Central, Western, Southern, Copperbelt, Lusaka, and North-Western), Namibia (Ohangwena and Okavango), and Angola (Cuando-Cubango, Moxico, Bie, Benguela, Cunene, Huambo, Huila, and Namibe).

#### Rural residence.

**Fig 4f** shows a weak inverse association between the proportion of children with zero-dose status and the proportion of mothers residing in the rural settings across regions in SSA after adjusting for other confounding factors. This relationship was consistent across all regions in SSA but weaker in West, Central and East African countries.

#### Female child.

**Fig 4g** shows a significant positive and inverse association between the proportion of zero-dose children and the proportion female children from Central and Southern Africa after adjusting for other variables. The inverse association was strong in Bie and Lunda Sul regions of Angola. Other regions in SSA did not show any significant findings.

#### No employment.

**Fig 4h** shows both significant positive and significant inverse associations between the proportion of children with zero-dose status and the proportion of mothers not currently working in areas of Central and Southern Africa. A positive but weak relationship was observed in Ennedi region of Chad. An inverse association was observed in Southern Africa but this relationship appeared to be stronger in some sub-regions of Angola (Benguela, Cunene, Huila and Namibe), and Namibia (Erongo, Khomas, Kunene, Ohangwena, Omusati, Oshana, Oshikoto and Otjozondjupa). No significant findings were observed in other regions in SSA.

##### Low birth weight.

**Fig 4i** shows an inverse association between the proportion of children with zero-dose status and the proportion of children with low birth weight in Angola (Bie, Lunda Norte, Lunda Sul, Malange and Moxico), with no significant findings in other regions studied.

#### Mass media.

**Fig 4j** shows a weak association between the proportion of zero-dose children and proportion of mothers with no media access in some parts of West Africa after adjusting for other variables. Other regions did not show a significant association.

## Discussion

This multi-country study examined the geographical clustering of zero-dose children aged 12–59 months in SSA and assessed the sociodemographic characteristics associated with pattern of clustering among regions in SSA, using multiscale geographically weighted regression analysis. Importantly, this approach provides localized insights into spatial heterogeneity and allows relationships between zero-dose prevalence and theoretically important sociodemographic characteristics to vary across administrative units.

We found overall high rates of zero-dose children in SSA with clear evidence of spatial clustering in some areas. Across 33 SSA countries, the mean prevalence of zero-dose status among children aged 12–59 months is 6.8%, aligning with an estimate of 9.9% from a previous study on lower middle-income countries focused on children in the same age group [33]. We identified 27 regions in 8 SSA countries with significantly high zero-dose rates clustered as hot-spots. Most high-high clusters were observed in seven Western and Central African countries (Angola, Cameroon, Chad, Guinea, Mali, Niger, and Nigeria), with only one country in Eastern Africa (Zimbabwe) having high-high clusters. These patterns are consistent with recent work that documents generally higher prevalence of zero-dose children in Central and West Africa compared to East and Southern Africa [34]. Prior studies have attributed higher prevalence of underimmunized and zero-dose children to both individual and place-based factors, including maternal health-seeking behavior, low levels of domestic health investment and, particularly in the Sahal region of Africa, ongoing conflicts, political instability, and displacement [34,35]. To our knowledge, this is the first study to utilize geographically weighted models to examine the spatial heterogeneity of zero-dose children in SSA.

We also considered sociodemographic factors that may correlated with zero-dose status. Overall, we found that regions with higher proportions of adolescent mothers, mothers with no ANC visits, mothers who delivered at home, or mothers with no access to mass media and regions with lower proportions of children residing in the rural areas, on average, had higher proportions of zero dose children. However, we uncovered significant spatial variability in these relationships.

One pattern was consistent across SSA: regions with higher proportions of mothers with no ANC visits were found to have high rates of zero-dose children. This result is consistent with previous studies in SSA, indicating that children whose mothers attended four or more ANC visits were more likely to have timely and complete childhood vaccinations [36–38]. The value of ANC is evident, providing essential information on routine immunizations for children and facilitating access to other essential healthcare services for mothers [39].

Contrary to our expectations, regions with higher proportions of rural residents were generally found to have lower proportions of children with zero-dose status. This finding conflicts with numerous reports indicating that children in rural areas are more likely to miss basic childhood vaccinations and are at increased risk of being unvaccinated (zero-dose) compared to their counterparts in the urban areas [10,40,41]. However, some studies in SSA have reported that children from urban areas were more likely to be unimmunized [22,42]. This finding warrants further investigation to understand the underlying reasons.

Other relationships varied (in terms of strength and/or direction) across SSA. For example, in West, Central, and Southern Africa, regions with a higher proportion of mothers with no education were found to have a greater proportion of zero-dose children, consistent with OLS estimates from prior studies in West Africa [43,44], multi-country studies [42,45], and a systematic review and meta-analysis [46]. This positive association may be linked to the fact that educated mothers are more knowledgeable about the importance of immunization which helps them to make vital health decisions for their children. Of note, we found that the relationship between maternal education and zero dose status was reversed in East Africa, where regions with high proportions of uneducated women were found to have low prevalence of zero-dose children. This counterintuitive finding contradicts previous evidence from Ethiopia which associated lack of education with unimmunized children [47]. This finding warrants further investigation, given inadequate education of women and girls in SSA.

A significant relationship between home delivery and zero-dose status was unique to certain regions in South and East African countries. More specifically, regions with a high proportion of women who delivered at home, including regions in Angola, Namibia, South Africa, Lesotho, Zambia, Malawi, Mozambique, and Zimbabwe, were found to have higher proportions of zero-dose children. This might be attributed to different strategies promoting maternal and child health in these regions, including the use of traditional birth attendants and retired midwives, which serve as essential links to healthcare [47–51]. Our findings align with previous research from SSA, where hospital delivery was associated with improved childhood vaccination rates [38,52].

Religion also appears to play a role in childhood vaccination status, particularly in West, Central, and East African regions. Across these regions of SSA, the proportion of Muslim mothers was positively associated with the proportion of zero-dose children. This finding aligns with published studies from sub-Saharan African countries, highlighting religion as an important predictor of immunization uptake [53–56].

The study also revealed significant local variability in the relationship between maternal age and zero-dose status. In select regions of Senegal, Gambia, Guinea, Congo, Zambia, Namibia, and Angola, the proportion of adolescent mothers was positively associated with the proportion of children with zero-dose status. However, across all regions in Chad and in some regions in Niger, a higher proportion of adolescent mothers was associated with lower zero-dose prevalence, indicating an inverse relationship. Previous multi-country studies of populations across SSA have generally found that children of younger mothers have increased odds of being unimmunized [34,42]; however, these studies did not consider cross-national differences or local variability in this aggregate relationship. The inverse relationship observed in regions of Chad and Niger, which to our knowledge has not previously been reported, underscores the value of spatial regression techniques to reveal distinct local patterns and also highlights the need for further research to better understand the contextual factors that may help explain the reduced risk of zero-dose status among children of younger mothers in these areas.

Spatial variation was also evident in the relationship between maternal employment and zero-dose status. In the Ennedi region in Chad, a higher proportion of mothers who were not currently working was weakly associated with higher zero-dose prevalence. This pattern was reversed in Southern Africa, including in regions of Angola, Namibia, and South Africa. Previous multi-country studies of populations in SSA have found that children of mothers who are not currently working have increased odds of being unimmunized [24,42], though the strength of this relationship may be attenuated after adjusting for community-level unemployment rates [34]. Research exploring variation in this relationship across countries or subnational regions is limited. An analysis of data from the South African DHS provides some suggestive evidence consistent with an inverse relationship: the odds of missed opportunities for vaccination may be lower for children whose mothers are not working, though this association did not meet the threshold for statistical significance [57]. Taken together, these findings point to a need for further research into how maternal employment relates to zero-dose status across varying contexts.

### Strengths and Limitations

A major strength of this study is the application of advanced spatial regression techniques to capture spatial heterogeneity in the prevalence of zero-dose children in SSA. While MGWR has been used to uncover spatial variability in other important public health outcomes [15,16], this approach is yet to be applied in the context of zero-dose populations. We present what we believe to be the first, in-depth examination of how relationships between zero-dose prevalence and theoretically important sociodemographic factors in SSA vary over space. The use of separate bandwidths for each covariate further enhances our understanding of spatial associations, enabling more targeted interventions. A second strength of our study is the utilization of nationally representative data from 33 countries, which serves to strengthen the generalizability of study findings and enhance their usefulness for informing interventions in SSA. Finally, the study is well-aligned with recent initiatives including the IA2030 and SDG, aimed at reducing the number of zero-dose children by 2030.

This study is not without limitations. First, given the ecological nature of the study, ecological fallacy cannot be overlooked. Inferences can only be made at the level of spatial units examined (regions), and not at the level of individual participant. Second, spatial analysis is sensitive to the scale of spatial aggregation. Different choices of spatial scale could influence the observed patterns and results. Third, the DHS datasets used in this study correspond to surveys conducted over the period 2010–2020. This temporal variability could affect direct comparisons and interpretations of spatial clustering, particularly for countries with older survey data as findings may reflect past conditions that have since evolved. Fourth, due to the cross-sectional nature of the data, this study cannot establish causality and findings should be interpreted as associations. Fifth, while the DHS prioritizes vaccination card data to determine a child’s vaccination status, parental recall is used when a card is unavailable, which may introduce recall bias. Finally, there may be additional unaccounted factors, including factors unobserved in the DHS, that contribute to regional variation in the prevalence of zero-dose children status in SSA. Nonetheless, study findings can provide valuable guidance for policymakers in designing effective strategies to reduce the number of zero-dose children and improve overall immunization rates across SSA.

### Policy implications

Across countries and subnational regions, a higher proportion of mothers with no ANC visits was consistently and strongly associated with higher zero-dose prevalence. Policies that promote ANC utilization not only improve maternal health outcomes but also create opportunities to reach children who might otherwise remain unvaccinated. Given that many women in SSA continue to seek care from traditional birth attendants (TBAs), governments could consider strategies to formally train and integrate TBAs within the broader healthcare system. Such collaboration could enable TBAs to serve as trusted referral agents, linking mothers and newborns to accredited health facilities and helping to bridge the gap between formal and informal care [49–51,58].

In contrast to the consistently observed relationship between ANC visits and zero-dose prevalence, our analysis uncovered substantial spatial variability in most other associations. This heterogeneity suggests that national-level immunization strategies alone are unlikely to address the localized determinants of zero-dose status in SSA. Instead, these patterns underscore the need for context-specific, data-driven interventions. In the context of Nigeria, for example, the proportion of Muslim mothers was positively associated with the proportion of children with zero-dose status, specifically in the North-West and North-East regions. In these areas, culturally sensitive outreach that actively engages traditional and religious leaders (e.g., emirs, imams, and district heads) is recommended [59]. This association was not observed in the South-West region. In areas of the South-West, where the proportion of mothers with no education was positively associated with the proportion of children with zero-dose status, community- or facility-based health education interventions aimed at improving knowledge about immunization benefits and efficacy should be prioritized [60].

## Conclusion

Using multiscale geographically weighted regression analysis, this study provides clear evidence of spatial heterogeneity in the prevalence of zero-dose children in SSA as well as local variation in the relationships between theoretically important sociodemographic factors and zero-dose prevalence. While progress has been made in expanding immunization coverage globally, SSA still faces challenges related to zero-dose children, putting the region at risk of potential disease surges such as measles and polio outbreaks. The global focus on leaving no one behind, as demonstrated by Gavi’s 5.0 strategy and the IA2030 goals, aligns with the commitment to reduce the number of zero-dose children and prevent preventable deaths of newborns and under-five children by 2030.

Addressing the issue of zero-dose children is crucial for safeguarding public health and achieving the broader goals of disease eradication and child well-being. Targeted interventions directed towards the underserved and hard-to-reach communities are needed to reduce the number of zero-dose children in SSA. Collaboration between government and non-governmental organizations, along with the adoption of evidence-based practices to ensure vaccines reach marginalized communities, is needed to effectively address this issue.

## Supporting information

S1 Fig Prevalence of zero-dose children across SSA. Two categories defined relative to mean prevalence of zero-dose children across SSA (6.8%). First-level subdivision boundary shapefiles were downloaded from the GADM website (https://gadm.org/).
(TIF)

S2 Fig Distribution of explanatory variables across SSA. Five categories defined by quintiles. First-level subdivision boundary shapefiles were downloaded from the GADM website (https://gadm.org/).
(TIF)
