# Supplementary figures and images for: Spatial clustering of zero dose children aged 12 to 59 months across 33 countries in sub-Saharan Africa: A multiscale geographically weighted regression analysis
Source: PLoS One. 2025 Dec 10;20(12):e0338568. doi: 10.1371/journal.pone.0338568 (PMC12694887; doi:10.1371/journal.pone.0338568)

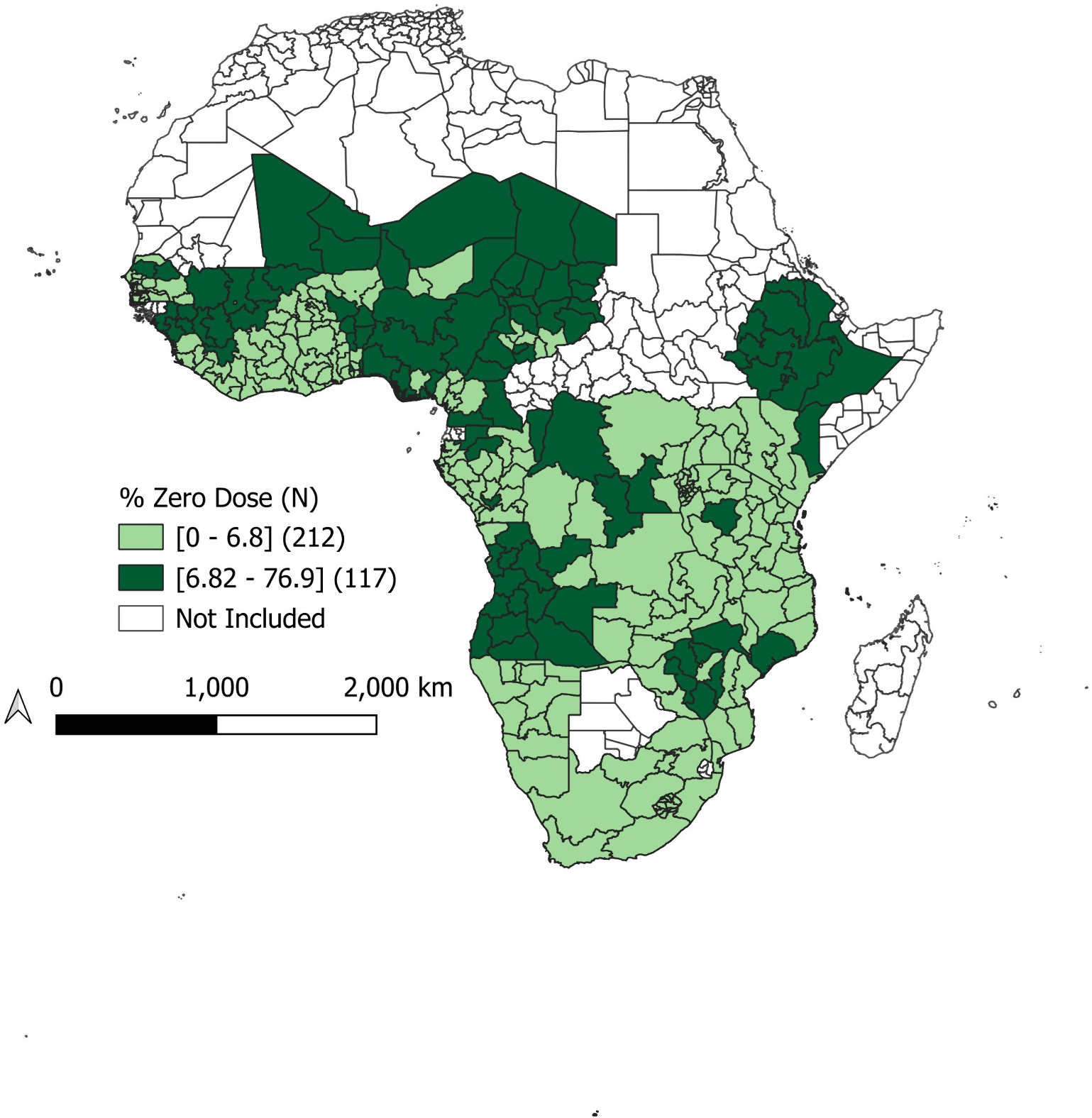

Supplement: S1 Fig — Two categories defined relative to mean prevalence of zero-dose children across SSA (6.8%). First-level subdivision boundary shapefiles were downloaded from the GADM website (https://gadm.org/). (TIF) [file pone.0338568.s001.tif]

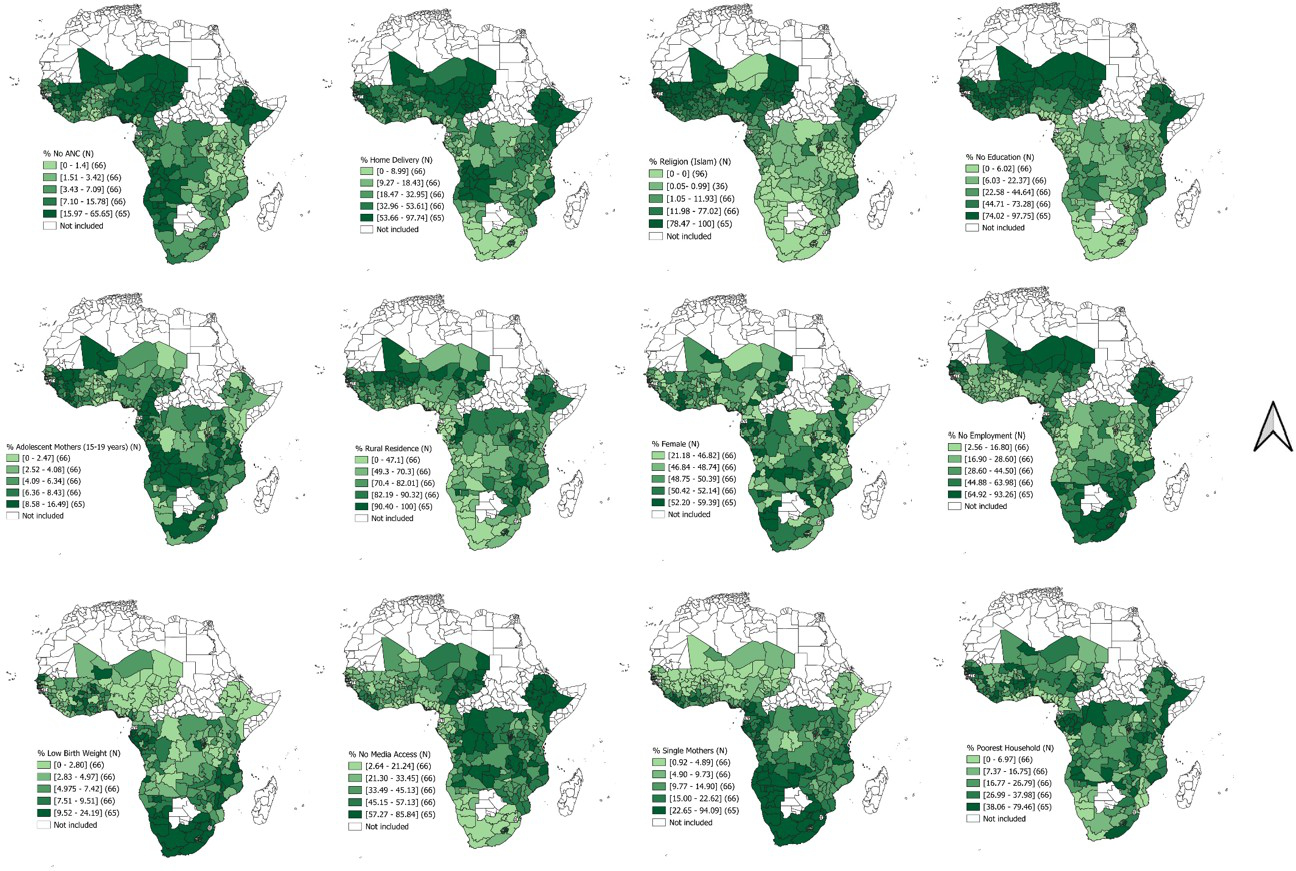

Supplement: S2 Fig — Five categories defined by quintiles. First-level subdivision boundary shapefiles were downloaded from the GADM website (https://gadm.org/). (TIF) [file pone.0338568.s002.tif]
